# Clinical trials for authorized biosimilars in the European Union: a systematic review

**DOI:** 10.1111/bcp.13076

**Published:** 2016-09-05

**Authors:** Johanna Mielke, Bernd Jilma, Franz Koenig, Byron Jones

**Affiliations:** ^1^ Statistical MethodologyNovartis Pharma AG4056BaselSwitzerland; ^2^ Department of Clinical PharmacologyMedical University of Vienna1090ViennaAustria; ^3^ Center for Medical Statistics, Informatics and Intelligent SystemsMedical University of Vienna1090ViennaAustria

**Keywords:** biosimilar drug development programmes, biosimilarity, biosimilars, EMA, EPAR, trial design

## Abstract

**Aim:**

In 2006, Omnitrope (by Sandoz) was the first approved biosimilar in Europe. To date, 21 biosimilars for seven different biologics are on the market. The present study compared the clinical trials undertaken to obtain market authorization.

**Methods:**

We summarized the findings of a comprehensive review of all clinical trials up to market authorization of approved biosimilars, using the European public assessment reports (EPARs) published by the European Medicines Agency (EMA). The features compared were, among others, the number of patients enrolled, the number of trials, the types of trial design, choice of endpoints and equivalence margins for pharmacokinetic (PK)/pharmacodynamic (PD) and phase III trials.

**Results:**

The variability between the clinical development strategies is high. Some differences are explainable by the characteristics of the product; if, for example, the PD marker can be assumed to predict the clinical outcome, no efficacy trials might be necessary. However, even for products with the same reference product, the sample size, endpoints and statistical models are not always the same.

**Conclusions:**

There seems to be flexibility for sponsors regarding the decision as to how best to prove biosimilarity.

## Tables of Links

**Table:** 

**TARGETS**	
**Catalytic receptors** 2	**Enzymes** 3
Erythropoietin receptor	Insulin receptor
Growth hormone receptor	**G protein‐coupled receptors** 4
Tumour necrosis factor receptor	Follicle stimulating hormone receptor
Granulocyte colony‐stimulating factor	

**LIGANDS**	
Erythropoietin	Filgrastim
Follitropin	Insulin glargine
Etanercept	Growth hormone
Infliximab	

These Tables list key protein targets and ligands in this article that are hyperlinked to corresponding entries in http://www.guidetopharmacology.org, the common portal for data from the IUPHAR/BPS Guide to PHARMACOLOGY 1, and are permanently archived in the Concise Guide to PHARMACOLOGY 2015/16 2, 3, 4.

## Introduction

Biologics have revolutionized treatment in important disease areas, such as cancer, diabetes and inflammatory diseases. The downside of the use of biologics is the high cost; in 2002, $46 billion were spent on biologics worldwide and it is expected that this will increase to $221 billion in 2017 5.

Due to the high prices and the first expiry of patents of biologics over the last few years, the development of copies of biologics, so‐called biosimilars, has recently gained much attention. The European Medicines Agency (EMA) is the leading regulator in this regard, having approved the first biosimilar in 2006 (Omnitrope, by Sandoz), and since then, the landscape of authorized biosimilars in Europe has widened considerably. Currently, there are 21 products for seven different biologics on the market. It is predicted that the use of biosimilars may lead to a $250 billion reduction in spending on biologics in the US between 2014 and 2024 6.

So far, there is no unified definition of biosimilars that is accepted by all regulatory agencies. The EMA 7 defines a biosimilar as: ‘a biological medicinal product that contains a version of the active substance of an already authorized original biological medicinal product (reference medicinal product). A biosimilar demonstrates similarity to the reference medicinal product in terms of quality characteristics, biological activity, safety and efficacy based on a comprehensive comparability exercise’. There is also a need to distinguish between biologics and nonbiological complex medicinal products, as described recently 8.

Unlike chemically defined ‘simple’ molecules, as usually employed in e.g. oral generics, biosimilar active substances do not meet this general definition of the ‘same active substance’ because some characteristics are very sensitive to the manufacturing process, which cannot be completely duplicated. This was also stated by the Directive 2001/83/EC of the European Parliament and of the Council (Article 10) 9. The more complex structure and the larger molecule size of biologics makes them more complicated to develop. This complexity results in an extended approval process, which involves large clinical trials in addition to a comprehensive analytical and nonclinical analysis. In contrast to the approval process for generics, for which showing bioequivalence of pharmacokinetic (PK) parameters is considered most sensitive to substantiate therapeutic equivalence, in most cases phase III trials are additionally demanded for showing biosimilarity 10. From a regulatory perspective, biosimilars are therefore not accepted as generic applications.

This particularity has also led the EMA to consider that the standardized approach that is used for generics is not feasible for biosimilars 7. Therefore, it can be expected that the development programmes of the drug sponsors will differ. Wang and Chow 11 compared the properties of the PK and pharmacodynamic (PD) studies involved in the approval of the six biosimilars that were available at that time. They found considerable variability in the equivalence assessments – e.g. the amount of PD analysis needed ranged from no analysis at all to complex analyses involving formal testing procedures. However, the focus of their paper was on PK and PD studies only. Several review papers have also focused on biologics that are biosimilar to a specific active substance – e.g. on epoetin 12, 13, 14, 15, filgrastim 16 and infliximab 17. To our knowledge, no overall comparison of the clinical development programmes for all 21 marketed biosimilars has yet been published. In the current review, we present the results of a comparison of the PK, PD and phase III trials for all marketed biosimilars in Europe.

## Methods

The main resources for this project were the European public assessment reports (EPARs) that were published by the EMA 18 and are available online 19, 20, 21, 22, 23, 24, 25, 26, 27, 28, 29, 30, 31, 32, 33, 34, 35, 36, 37, 38, 39. If not stated otherwise, the information presented here is taken from these reports. Only studies up to the date of market authorization were considered; postmarketing commitments were not analyzed.

If information was missing in the EPARs or details of the studies were not clear, an online search was conducted using PubMed, ISI's web of science, clinicaltrials.gov, EudraCT and Google Scholar, using keywords such as the drug name and the sponsor, the international nonproprietary name and the sponsor or the trial identifier of the sponsor.

The properties of the submitted studies were compared with those described in the available EMA guidelines for the assessment of biosimilarity 7, 40 and with the product‐specific guidelines. The product‐specific guidelines take into account the characteristics of the substance and give detailed recommendations for the trial design 41.

The present study analyzed the clinical trials of authorized biosimilars only. Neither products that were withdrawn before the decision of the Committee for Medicinal Products for Human Use (CHMP) (e.g. Isomarv, Marvel LifeSciences Ltd 42) nor products that were withdrawn after market authorization (e.g. Filgrastim ratiopharm, Ratiopharm GmbH 43) were considered. Products for which the CHMP refused approval were also not analyzed. At the time of the study, this was the case for two products: Solumarv (Marvel Lifesciences Ltd 44) and Alpheon (BioPartners GmbH 45). This left a total of 21 applications of biosimilars to be considered.

Not all of these 21 biosimilars are, in fact, different products; some companies conducted a joint development programme but marketed a product under different brand names. For example, for the active substance infliximab, Stada Arzneimittel AG and Hospira UK Ltd submitted a joint application. This product is sold by Stada Arzneimittel AG as Silapo, whereas the Hospira UK Ltd product is called Retacrit. As the submitted clinical trials were identical, the application was considered as a single project in the current study, and the different products are separated with a slash – e.g. Silapo/Retacrit.

For assessing the number of trials, it should be noted that if a trial had an extension using exactly the same set of patients, this was not counted in the current study as a separate trial. Studies that were listed in the EPAR but did not involve the test product (e.g. studies comparing a US and an EU product – i.e. for the biosimilars Benepali and Abasaglar) were not considered in the current study.

## Results

At the time of the study, there were 21 biosimilars (resulting from 13 different applications) on seven different biologics available on the European Market. Table 1 shows an overview of the reference products for the approved biosimilars and their mechanisms of action. These drugs act endocrinologically, to stimulate body growth or oozyte maturation, and include insulin‐like growth factor to boost erythropoiesis or granulopoiesis, and the more recently developed immunosuppressive antibodies that inhibit the cytokine tumour necrosis factor‐alpha.

**Table 1:** Overview of biologics for which a biosimilar is authorized in Europe

**Active substance**	**Originator drug name**	**Originator company**	**Mechanism of action**
**Haematopoietic growth factors**			
**Epoetin alfa/zeta**	Eprex (EU), Erypo (Germany)	Janssen/Ortho Biotech	The active substance, epoetin alfa, is a copy of a hormone called erythropoietin, and works in exactly the same way as the natural hormone to stimulate the production of red blood cells from the bone marrow
**Filgrastim**	Neupogen	Amgen/Roche	Filgrastim acts in the same way as naturally produced granulocyte colony‐stimulating factor by encouraging the bone marrow to produce more white blood cells
**Endocrinologically acting drugs**			
**Follitropin alfa**	Gonal‐f	Merck Serono Europe	The active substance in Gonal‐f, follitropin alfa, is a copy of the natural hormone, follicle‐stimulating hormone (FSH). In the body, FSH controls reproductive function: in women, it stimulates the production of eggs; and in men, it stimulates the production of sperm in the testicles
**Insulin glargine**	Lantus	Sanofi‐Aventis	This is a replacement insulin that is very similar to the insulin made by the body. The replacement insulin acts in the same way as naturally produced insulin and helps glucose to enter cells from the blood
**Somatropin**	Genotropin	Pfizer	This promotes growth during childhood and adolescence, and also affects the way that the body handles proteins, fat and carbohydrates. The active substance, somatropin, is identical to the human growth hormone
**Anti‐inflammatory blockers of tumour necrosis factor alpha**			
**Etanercept**	Enbrel	Pfizer	The active substance, etanercept, is a protein that has been designed to block the activity of a chemical messenger in the body called tumour necrosis factor (TNF)
**Infliximab**	Remicade	Janssen	The active substance in Remicade, infliximab, is a monoclonal antibody. A monoclonal antibody is an antibody (a type of protein) that has been designed to recognize and attach to a specific structure (called an antigen) in the body. Infliximab has been designed to attach to a chemical messenger in the body called TNF‐alpha. This messenger is involved in causing inflammation and is found at high levels in patients with the diseases that Remicade is used to treat. By blocking TNF‐alpha, infliximab improves the inflammation and other symptoms of these diseases

The mechanism of action is quoted with only minor modifications from the ‘EPAR – Summaries for the public’ available in the EPAR database of EMA 18

### Indications and extrapolation

In most cases, the indications applied for are essentially the same as those of the reference product. Some restrictions were stated when considering the active substances epoetin alfa and epoetin zeta, which are both types of erythropoietin; for Silapo/Retacrit (biosimilar to epoetin zeta), the indication of ‘reduction in allogeneic blood transfusions in adult non‐iron‐deficient patients prior to major elective orthopaedic surgery’ was not granted owing to the lack of shown equivalence for the subcutaneous (SC) administration route. Epoetin Alfa Hexal/Abseamed/Binocrit (biosimilar to epoetin alfa) is not indicated for ‘increasing the yield of autologous blood from patients in a predonation programme’. More detailed information on the indications is available in Table S1.

In most cases, only one phase III trial in one indication was submitted for authorization and it was assumed that the results could be generalized to all other indications with the same route of administration. For example, for Remsima/Inflectra (active substance infliximab), the phase III trial was undertaken in patients with active rheumatoid arthritis 46, but the product is licensed for all indications of the reference product, including rheumatoid arthritis, ankylosing spondylitis, psoriatic arthritis, psoriasis, adult and paediatric Crohn's disease, and adult and paediatric ulcerative colitis. In the EPAR, it is stated that the sponsor provided a literature review of the therapeutic indication and also of the mechanism of action of the drug. According to the report, the sponsor also presented preliminary data on 23 patients with either Crohn's disease or ulcerative colitis (no details were given in the EPAR) and agreed to conduct additional postmarketing studies. Using all submitted information, the regulators allowed the company an extension to all indications. More background information about the extrapolation of Remsima/Inflectra can be found in the study by Reinisch *et al*. 47.

Although two active substances (filgrastim, somatropin) received orphan drug designations for specific indications 48, none of the biosimilars had orphan drug status according to the EPAR database of the EMA 18.

### PK/PD‐ *vs.* phase III trials

Table 2 shows an overview of the study populations enrolled both in the PK or PD trials and in the phase III trials. A trial was counted as a PK/PD trial in the current study only if the primary endpoint was a measure of PK or PD. A trial with efficacy as the primary endpoint and additional PK assessments was therefore not listed as a PK/PD trial.

**Table 2:** Population size and number of trials for assessing biosimilarity

**Active substance**	**Product**	**Company**	**Number of subjects in PK/PD studies** **(P: patients, V: volunteers)**	**Number of PK/PD studies**	**Number of patients in phase III studies**	**Number of phase III studies**	**Compared with reference product in phase III studies?**
**Epoetin alfa/zeta**	Silapo/Retacrit	Stada Arzneimittel AG/ Hospira UK Limited	72 (V)	2	1272	3	Yes
	Epoetin Alfa Hexal/Abseamed/Binocrit	Hexal/Medice Arzneimittel Puetter/Sandoz	234 (V)	5	592	2	Yes
**Filgrastim**	Zarzio/Filgrastim Hexal	Sandoz/Hexal	146 (V)	4	170	1	No
	Tevagrastim/Ratiograstim/Biograstim	Teva Generics/Ratiopharm/ABZ‐Pharma	200 (V)	2	677	3	Yes
	Nivestim	Hospira UK Ltd	92 (V)	2	279	1	Yes
	Grastofil/Accofil	Apotex Europe BV/Accord Healthcare	215 (V)	4	120	1	No
**Follitropin alfa**	Ovaleap	Teva Pharma	76 (V)	2	299	1	Yes
	Bemfola	Finox Biotech	24 (V)	1	273	1	Yes
**Insulin glargine**	Abasaglar	Eli Lily	211, 20 (V, P)	5	1295	2	Yes
**Somatropin**	Omnitrope	Sandoz	61 (V)	3	140	2	Yes
**Etanercept**	Benepali	Samsung Bioepis UK Limited	138 (V)	1	596	1	Yes
**Infliximab**	Remsima/Inflectra	Celltrion Healthcare/Hospira UK Limited	269 (P)	2	606	1	Yes
	Flixabi	Samsung Bioepis UK Limited	159 (V)	1	584	1	Yes

PD, pharmacodynamic; PK, pharmacokinetic. All information is taken from the EPARs 19, 20, 21, 22, 23, 24, 25, 26, 27, 28, 29, 30, 31, 32, 33, 34, 35, 36, 37, 38, 39

**Figure 1:**
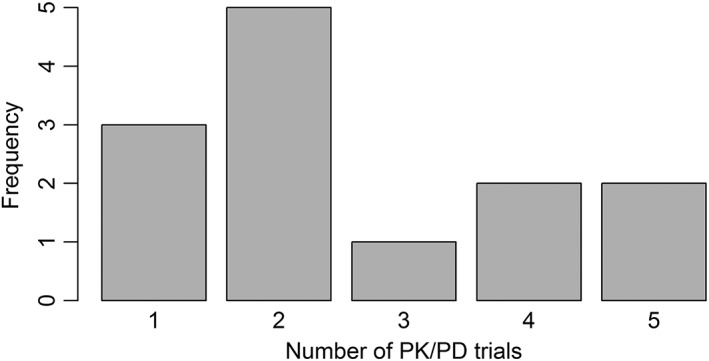
Overview of the number of pharmacokinetic/pharmacodynamic (PK/PD) trials undertaken for obtaining approval as a biosimilar. A trial is counted as a PK/PD trial if the primary endpoint is PK or PD

The sponsors conducted between one and five PK/PD trials with, in total, 24–269 patients involved. This is shown in Figure 1. The PK/PD trials were mostly undertaken in healthy volunteers; for the application of Remsima/Inflectra, only patients were used, and for insulin glargine, there were 20 patients enrolled, in addition to 211 volunteers. For insulin glargine, the additional use of patients was requested by the CHMP according to the EPAR. While no specific reasons are mentioned in the publically available documents for Remsima/Inflectra, this product has specific and potentially severe adverse effects, which make extended testing of infliximab in healthy volunteers appear undesirable. For example, infliximab may induce anaphylaxis 49 and reactivate tuberculosis 50, 51.

Between one and three phase III trials were submitted, with between 120 and 1295 patients enrolled. Particularly small sample sizes were observed for Zarzio/Filgrastim Hexal (170 subjects), Grastofil/Accofil (120 subjects) and Omnitrope (140 subjects). While the applications for the first two were focused on PK/PD‐trials (see discussion below), the studies for the latter were done in children, which might explain the small sample size.

The relative size of the PK/PD trials in comparison with phase III trials also varied considerably – i.e. the ratio of the number of patients and volunteers in PK/PD trials to the number of patients in phase III trials ranged from 1.79 (for Grastofil/Accofil) to 0.06 (for Silapo/Retacrit). For the approval of Grastofil/Accofil (active substance filgrastim), 215 subjects took part in four PK/PD studies 52, whereas only 72 volunteers participated in two trials with Silapo/Retacrit (active substance epoetin zeta) 53, 54. By contrast, in phase III studies, the sponsors conducted three efficacy trials for Silapo/Retacrit, with more than 1000 patients, but only a single‐arm trial (no comparison with reference), with 120 subjects, for Grastofil/Accofil.

It should be taken into account that Silapo/Retacrit and Grastofil/Accofil have different reference products, with different indications. One explanation for the variation in sample size is that if the PD marker is predictive for the clinical efficacy outcome, the product‐specific guidelines allow the possibility of replacing larger phase III trials with PK/PD trials. The product‐specific guideline for Grastofil/Accofil is about products containing granulocyte colony‐stimulating factor 55. There, it is said in the context of the assessment of efficacy that: ‘alternative models, including PD studies in healthy volunteers, may be pursued for the demonstration of comparability if justified’. This will also be reiterated more specifically in the planned revision of that guideline 56.

However, there are also product‐specific guidelines describing the surrogate markers that should not be used for efficacy comparison. For example, the guidelines for products containing recombinant erythropoietins 57 mention that the recommended PD marker reticulocyte count ‘is not an established surrogate marker for the efficacy of epoetin and therefore not a suitable endpoint in clinical trials’.

Even for one active substance, the splitting of resources into phase III and PK/PD studies may not be the same, which is illustrated by the four filgrastim applications Grastofil/Accofil, Zarzio/Filgrastim Hexal, Tevagrastim/Ratiograstim/Biograstim and Nivestim. For Grastofil/Accofil and Zarzio/Filgrastim Hexal, only single‐arm trials were undertaken in phase III. Therefore, no direct head‐to‐head comparison of clinical efficacy with a reference product was performed. The applications for Tevagrastim/Ratiograstim/Biograstim and Nivestim contained comparative trials in phase III – e.g. three phase III trials, involving a total of 677 subjects, were carried out for Tevagrastim/Ratiograstim/Biograstim. Therefore, the EMA allows some flexibility for the sponsors to decide which approach they can choose for proving biosimilarity.

### Trial design

Table 3 shows the details of the submitted studies for all products. It indicates the assessments for which they were used (efficacy, safety, PK, PD). A trial is considered as part of the assessments in these categories if the results of the trial are discussed in the relevant sections in the EPAR. As safety is evaluated in nearly all studies, we focused on the efficacy, PK and PD studies.

**Table 3:** Overview of study designs that were used in the clinical development programme for obtaining approval as a biosimilar. Only studies undertaken prior to market authorization are listed

**Active substance**	**Product**	**Study design**	***N***	**Single/multiple dose**	**Route of administration, dose**	**PK**	**PD**	**E**	**S**
**Epoetin alfa/zeta**	Silapo/Retacrit	2 × 2 crossover	24	Single	IV, dose n.s.	X	–	–	–
		3‐period crossover	48	Single	IV, SC, dose n.s.	X	–	–	–
		Parallel group^a^	609	Multiple	IV, 1000 IU or 2000 IU 3 t.i.w.^13^	–	–	X	X
		2 × 2 crossover^a^	402	Multiple	IV, 1000 IU or 2000 IU 1–3 t.i.w.	–	–	X	X
		Single‐arm^a^	261	Multiple	n.s., individual doses	–	–	X	X
	Epoetin Alfa Hexal/ Abseamed/Binocrit	2 × 2 crossover	6	Single	IV, SC, 100 μg kg^−1^	X	X	–	X
		Parallel group	76	Multiple	IV, 100 μg kg^−1^ 3 t.i.w.	X	X	–	X
		Parallel group	74	Multiple	SC, 100 μg kg^−1^ 3 t.i.w.	X	X	–	X
		Test *vs*. NeoRecormon (epoetin beta), trial design not given	72	Single, multiple	SC, 100 μg kg^−1^ 3 t.i.w.	–	–	–	–
		Single‐arm	6	Multiple	SC,100 μg kg^−1^ 3 t.i.w.	–	–	–	–
		Parallel group^a^	478	Multiple	IV, 3 t.i.w., dose before randomization^14^	–	–	X	X
		Parallel group^a^	114	Multiple	SC, dose n.s.	–	–	X	X
**Filgrastim**	Zarzio/Filgrastim Hexal	2 × 2 crossover	40	Multiple	SC, 10 μg kg^−1^ day^−1^	X	X	–	X
		2 × 2 crossover	26	Single	IV, 5 μg kg^−1^ day^−1^	X	X	–	X
		2 × 2 crossover	56	Multiple	SC, 2.5 μg kg^−1^ day^−1^ or 5 μg kg^−1^ day^−1^	X	X	–	X
		2 × 2 cross‐over	24	Single	SC, 1 μg kg^−1^ day^−1^	X	X	–	X
		Single‐arm^a^	170	Multiple	SC, 30 MIU for <60 kg, 48 MIU for ≥60 kg	–	–	X	X
	Tevagrastim/Ratiograstim/Biograstim	2 × 2 crossover	56	Single	SC, 5 μg kg^−1^ or 10 μg kg^−1^	X	X	–	X
		2 × 2 crossover	144	Single	IV with 5 μg kg^−1^ or 10 μg kg^−1^ or SC with 5 μg kg^−1^ or 10 μg kg^−1^	X	X	–	X
		Placebo and active control parallel group^1,17,^ a	348	Multiple	SC, 5 μg kg^−1^ day^−1^	X	–	X	X
		Parallel group in first cycle, switch to test afterwards^2,^ a	237	Multiple	SC, 5 μg kg^−1^ day^−1^	X	–	X	X
		Parallel group in first cycle, switch to test afterwards^3,^ a	92	Multiple	SC, 5 μg kg^−1^ day^−1^	X	–	X	X
	Nivestim	2 × 2 crossover	44	Single	IV or SC, 10 μg kg^−1^ day^−1^	X	X	–	X
		2 × 2 crossover	48	Multiple	SC, 5 μg kg^−1^ day^−1^ or 10 μg kg^−1^ day^−1^	X	X	‐	X
		Parallel group^4,^ a	279	Multiple	SC, 5 *μg* kg^−1^/day	–	–	X	X
	Grastofil/Accofil	2 × 2 crossover	35	Single	IV, 5 μg kg^−1^	X	X	–	X
		2 × 2 crossover	68	Single	SC, 75 μg or 150 μg	X	X	–	X
		Placebo and active control parallel group	69	Multiple	SC, 5 μg kg^−1^	X	X	–	X
		3‐period crossover (EU reference *vs*. US reference *vs*. test)	43	Single	SC, 300 μg	X	X	–	X
		Single‐arm^5,^ a	120	Multiple	SC, 5 μg kg^−1^	–	X	X	X
**Follitropin alfa**	Ovaleap	Single‐arm	40	Single	SC, 37.5 IU, 75 IU, 150 IU or 300 IU	X	–	–	X
		2 × 2 crossover	36	Single	SC, 300 IU	X	–	–	X
		Parallel group^6,^ a	299	Multiple	SC, start dose: 150 IU^15^	–	X	X	X
	Bemfola	2 × 2 crossover	24	Single	SC, 225 IU	X	–	–	X
		Parallel group^7,^ a	273	Multiple	SC, start dose: 150 IU^15^	–	X	X	X
**Insulin glargine**	Abasaglar	4‐period crossover (no details on sequences)	80	Single	SC, 0.5 U kg^−1^	X	X	–	X
		4‐period crossover (no details on sequences)	91	Single	SC, 0.5 U kg^−1^	X	X	–	X
		2 × 2 crossover	16	Single	SC, 0.5 U kg^−1^	X	X	–	X
		4‐period crossover (test and reference in two different doses, no information on sequences)	24	Single	SC, 0.3 U kg^−1^ and 0.6 U kg^−1^	X	X	–	X
		2 × 2 crossover	20	Single	SC, 0.3 U kg^−1^	–	X	–	X
		Parallel group^8,^ a	536	Multiple	SC, previous dose	–	–	X	X
		Parallel group^9,^ a	759	Multiple	SC, previous dose	–	–	X	X
**Somatropin**	Omnitrope	2 × 2 crossover (Somatropin Sandoz powder *vs*. placebo)	12	Single	SC, 5 mg	X	X	–	–
		2 × 2 cross‐over (Somatropin Sandoz powder *vs*. US reference)	25	Single	SC, 5 mg	X	X	–	–
		2 × 2 crossover (Somatropin Sandoz *vs*. Somatropin Sandoz Liquid)	24	Single	SC, 5 mg	X	X	–	–
		Parallel group^18,^ a	89	Multiple	SC, 0.1 IU kg^−1^ day^−1^	X	–	X	X
		Single‐arm^a^	51	Multiple	SC, 0.03 mg kg^−1^ day^−1^	–	–	X	X
**Etanercept**	Benepali	2 2 × 2 crossover (test *vs*. US reference, test *vs*. EU reference)	138	Single	SC, 50 mg	X	–	–	X
		Parallel group^10,^ a	596	Multiple	SC, 50 mg	X	–	X	X
**Infliximab**	Remsima/Inflectra	Parallel group	19	Multiple	IV, 3 mg kg^−1^	X	X	X	X
		Parallel group	250	Multiple	IV, 5 mg kg^−1^	X	–	X	X
		Parallel group^11,^ a	606	Multiple	IV, 3 mg kg^−1^	X	X	X	X
	Flixabi	3‐arm parallel group (test, EU reference, US reference)	159	Single	IV, 5 mg kg^−1^	X	–	–	X
		Parallel group^12,^ a	584	Multiple	IV, 3 mg kg^(−16)^	X	–	X	X

E, efficacy; IV, intravenous; *N*, number of subjects; n.s., not specified; PD, pharmacodynamic; PK, pharmacokinetic; S, safety; SC, subcutaneous; t.i.w., times a week; X, data from the study were discussed in this part of the EPAR

EudraCT‐ID: ^1^: 2004–001 452‐36, ^2^: 2004–001 450‐84, ^3^: 2004–001 449‐13, ^4^: 2007–000 394‐36, ^5^: 2007–005 034‐36, ^6^: 2009–017 674‐20, ^7^: 2010–019 287‐37, ^8^: 2011–000 829‐73, ^9^: 2011–000 828‐15, ^10^: 2012–005 026‐30, ^11^: 2010–018 646‐31, ^12^: 2012–005 733‐37

Further dosing details: ^13^: If a lower dose was needed it was given less frequently (e.g. twice or once every week). ^14^: Dose adjustments were allowed every three weeks. ^15^: Individual adjustments were possible. ^16^: Dose increments were allowed after week 30 by 1.5 mg kg^−1^ up to 7.5 mg kg^−1^ per visit

Further study design details: ^17^: After the first cycle, the placebo group switches to test; the primary endpoint was after cycle 1. ^18^: First part: Somatropin Sandoz powder *vs*. EU reference; second part: Somatropin Sandoz powder and Somatropin Sandoz Liquid; third part: Somatropin Sandoz Liquid

a Study is a phase III trial. All information is taken from the EPARs 19, 20, 21, 22, 23, 24, 25, 26, 27, 28, 29, 30, 31, 32, 33, 34, 35, 36, 37, 38, 39

In the 2 × 2 crossover design, each subject receives both test (T) and reference (R) products and is randomly assigned to receive the products either in the order RT or in the order TR, with the constraint that an equal number of subjects are assigned to each sequence. In a parallel group trial, each subject is randomly assigned to T or R whereas the number of subjects assigned to these categories do not necessarily need to be the same.

In the guideline on similar biological medicinal products containing biotechnology‐derived proteins as an active substance 40, it is recommended that a single‐dose 2 × 2 crossover trial be used for the assessment of PK and PD. This is in line with the bioequivalence guideline 58, which recommends that a randomized, single‐dose, crossover design (with sequence groups RT and TR, as above) is used to compare two formulations. Crossover trials have the advantage that the effect of confounding variables is reduced because every subject acts as his or her own control. This also leads to a more precise comparison of R and T, and, as a result, a smaller sample size as compared with a parallel group trial 59.

Most sponsors followed the advice in the above‐mentioned guideline and, in all but one case, at least one trial with a 2 × 2 crossover design was submitted. The only exceptions were the two biosimilars with the active substance infliximab, for which only parallel group trials were used. Nonetheless, this is in line with the product‐specific guideline on similar biological medicinal products containing monoclonal antibodies (mAbs), in which it is stated that: ‘a parallel group design may be necessary due to the long half‐life of mAbs and the potential influence of immunogenicity’ 60.

For Epoetin Alfa Hexal/Abseamed/Binocrit, the crossover trial consisted of only six subjects and was therefore not the main trial. The pivotal PK/PD trials were two parallel group trials, with a total of 150 volunteers. This contradicts the product‐specific guidelines on similar biological medicinal products containing recombinant erythropoietins, which recommend using single‐dose crossover trials 57. There is no comment in the EPAR or in the papers related to the trial 61, 62 on this deviation. However, it should be noted that the earliest product‐specific guideline on erythropoietins was published in 2006 63 and, as the drug was approved in 2007, the negotiation for the trial with the EMA most likely started before the guidance was released. In the application of Silapo/Retacrit (also a biosimilar to epoetin and approved in 2007), the PK/PD trials were conducted using a crossover design. Interestingly, one of the trials was not a 2 × 2 crossover but a three‐period, three‐treatment crossover design comparing the reference product after a single SC dose with the test product after intravenous (IV) and SC administration, which is a deviation from the standard approach. Therefore it seems that, at the time that the studies for both applications were planned, the sponsors had some flexibility in the choice of design.

In phase III, in most cases at least one parallel group design was used for comparing efficacy and safety. The only exception were the already discussed applications related to the active substance filgrastim. In this case, the guideline allowed the omission of an efficacy comparison in phase III. Therefore, some sponsors primarily used single‐arm trials in order to evaluate safety.

### Route of administration and single *vs*. multiple dose

For the route of administration in PK/PD trials, the overarching guideline 40 states that it is sufficient to use the SC route alone, although the reference product can be administered by both IV and SC routes. In the conducted trials, for all but the biosimilars with infliximab as active substance, at least one trial using the SC route of administration was performed. It should be noted that the reference product Remicade should only be administered by the IV route 64, so the assessment of the IV route of administration alone is fully justified.

The guidance 40 recommends a single‐dose administration. However, many sponsors also used multiple doses for their PK/PD assessment. In general, a multiple‐dose trial in patients is acceptable if a single‐dose trial cannot be conducted in healthy volunteers for tolerability reasons, and a single‐dose trial is not feasible in patients. This may have determined the trial design for Remsima/Inflectra. Alternatively, a signal in some endpoints can only be measured after repeated administration, such as the increase in CD34+ cell counts, which requires multiple doses of filgrastim 65.

### Endpoints, equivalence margins and statistical methods

Table 4 provides an overview of the endpoints and equivalence margins used for PK, PD and efficacy assessment. For PK and PD, the margins used were compared with the recommended margins in the product‐specific guidelines. The use of 90% confidence intervals for PK trials is recommended in the guidelines for the assessment of bioequivalence 58. For PD assessment, 95% confidence intervals were reported in the EPARs, which might be explained by the fact that PD parameters are already considered as efficacy parameters. Thus, the same standard as outlined in ICH E9 66 was applied as would be expected for any efficacy parameter in a clinical trial.

**Table 4:** Chosen endpoint and margins used in clinical trials

**Active substance**	**Product**	**PK endpoints**	**PK limits: 80–125%, CI: 90%**	**PD endpoints**	**PD limits: 80–125%, CI: 95%**	**Primary efficacy endpoint (type, measurement, margins)**
**Epoetin alfa/zeta**	Silapo/Retacrit	Yes	No (wider margins for Cmax)	–	–	Mean dosage [continuous, (−45, 45)]; mean haemogloblin level [continuous, (−1, 1) or (−0.6, 0.6)]
	Epoetin Alfa Hexal/Abseamed/Binocrit	Yes	Yes	Yes	No (tighter margins used)	Absolute change in haemogloblin levels between the screening/ baseline [continuous, (−0.5,0.5)]
**Filgrastim**	Zarzio/Filgrastim Hexal	Yes	Yes	Yes	No (tighter margins used)	Incidence (binary) and duration of severe neutropenia in cycle 1 in days (count) (−, −)
	Tevagrastim/Ratiograstim/Biograstim	Yes	Yes	Yes	Margins, yes; confidence level not given	The duration of severe neutropenia in cycle 1 in days (count) (−1 day, 1 day)
	Nivestim	Yes	Yes	Yes	Yes	The duration of severe neutropenia in cycle 1 in days (count) (−1 day, 1 day)
	Grastofil/Accofil	Yes	Yes	Yes	Yes	The duration of severe neutropenia in cycle 1 in days (count) (−, −)
**Follitropin alfa**	Ovaleap	No details	No details	X	No (only descriptive)	Number of oocytes retrieved [count, (−3, 3)]
	Bemfola	Yes	Yes	X	No (only descriptive)	Number of oocytes retrieved [count, (−2.9, 2.9)]
**Insulin glargine**	Abasaglar	Yes	Yes	Yes	Yes	Change in HbA1c from baseline to 24 weeks (continuous, non‐inferiority margin: 0.4%)
**Somatropin**	Omnitrope	Yes	Yes	Yes	No (no formal comparison, no results shown)	Height standardized by age and gender, Height velocity standard deviation score [continuous, (− 2.8, 2.8)]
**Etanercept**	Benepali	Yes	No details	–	–	ACR20 responders (binary, (−15%, 15%)
**Infliximab**	Remsima/Inflectra	Yes	Yes	X	No (no margins defined)	ACR20 responders (binary, (−15%, 15%))
	Flixabi	Yes	Yes	–	–	ACR20 responders (binary, (−15%, 15%))

Pharmacokinetic/pharmacodynamic (PK/PD) endpoints: are the endpoints used as described in the guidelines in at least one PK/PD study?; ACR, American College of Rheumatology; CI, confidence interval level; Cmax, maximum concentration; HbA1c, glycosylated haemoglobin; X = no recommendation given in guidelines, − = no assessment in application; (−, −) = no margins given. All information is taken from the EPARs 19, 20, 21, 22, 23, 24, 25, 26, 27, 28, 29, 30, 31, 32, 33, 34, 35, 36, 37, 38, 39

The endpoints and equivalence margins used for PK were mostly in line with the recommendations. Wider margins were used only for Silapo/Retacrit (code name SB309 in the EPAR) for the maximum concentration (Cmax) (70–143% instead of 80–125%). In the EPAR, it is explained that the sponsor ‘referred to the scientific advice given by CHMP in April 2004 that stated that the concept of “comparability” cannot use bioequivalence but that similar PK profiles of SB309 and the reference product would strengthen the choice of reference in the clinical trials. The advice concluded that for this purpose descriptive statistics will suffice’. Nonetheless, in the EPAR, 90% confidence intervals for the primary endpoints – area under the curve (AUC) and Cmax – were compared with the equivalence margins, which were, as stated in the EPAR, only defined *post hoc*. In the application for the other biosimilar to epoetin, Epoetin Alfa Hexal/Abseamed/Binocrit, the standard bioequivalence margins were used. Therefore, the deviation from the guidelines can, in this case, not be explained exclusively by the characteristics of the active substance.

For Ovaleap and Benepali, no margins and confidence levels were reported. Neither the EPAR nor the related publication 67 gives more than descriptive summary statistics for Ovaleap, so it is not clear if any formal testing was performed. In the EPAR for Benepali, it is stated that ‘the primary endpoints were well within the predefined acceptance range’, but neither confidence intervals nor acceptance ranges are stated.

Interestingly, even if the bioequivalence criteria are not fulfilled, the product might still be approved. For example, for Zarzio/Filgrastim Hexal, the sponsor submitted four PK/PD studies with a total of four different doses, one dose using both the IV and SC routes of administration (1 μg kg^−1^, 2.5 μg kg^−1^, 5 μg kg^−1^ and 10 μg kg^−1^ SC route; 5 μg kg^−1^ IV route). For the lower doses and after multiple SC doses, neither Cmax nor AUC met the acceptances ranges. In the EPAR, it is stated that: ‘the applicant claimed that the observed differences were due to differences in the levels of purity of the two products, leading to a systematic bias toward an apparently increased bioavailability for the reference product’. That is why the PK parameters were adjusted to the enzyme‐linked immunosorbent assay‐detectable dose and, consequently, the AUC then fulfilled the criterion. For Cmax, the values were still outside of the equivalence interval for a single dose of 2.5 μg kg^−1^ and multiple doses of both 2.5 μg kg^−1^ and 5 μg kg^−1^. The sponsor provided modelling results and an explanation of the mechanism of action. Overall, it was concluded that: ‘the small differences observed in the PK profile of filgrastim are not expected to translate into significant differences in the PD response’.

On the other hand, even if the equivalence criteria based on the confidence intervals are met, the CHMP might not be fully satisfied – e.g. for Grastofil/Accofil, the 90% confidence intervals for the ratios of all primary PK endpoints were in the predefined equivalence range of 80–125%. In the EPAR, it is acknowledged that the sponsor used the methods and equivalence limits requested by the CHMP. Nonetheless, as the AUC_0–32_ and the AUC_0–∞_ showed that the point estimates for test and reference were different, with a very low *P*‐value (*P* ≤ 0.0001) in two studies, the sponsor had to provide further analysis and justifications.

The recommendations concerning PD endpoints and equivalence margins are less concrete in the product‐specific guidelines than for PK. For two active substances (infliximab, follitropin alfa), no endpoints are named at all. In some product‐specific guidelines, it is mentioned that the known PD marker cannot predict the clinical outcome (described above for epoetin alfa/zeta). However, it is also stated that: ‘it is recommended that PD markers are added to the PK studies whenever feasible’ 40. Nonetheless, there are three applications without any PD assessment at all (Silapo/Retacrit, Benepali and Flixabi). However, these were applications which included large phase III trials with 1272, 596 and 584 patients, respectively. For Silapo/Retacrit, it was noted in the EPAR that the PD mechanism is known in the literature and it is reported that: ‘although PD studies should be part of the development programme for a biosimilar epoetin, the lack of such studies is not critical since demonstration of similar efficacy and safety between the new and the reference product is required anyway’. Therefore, it seems to be possible to get approval if the guidelines are not followed in detail, if the overall application is convincing.

In all cases in which advice in product‐specific guidelines was given and PD was evaluated in the application, the recommended endpoints were used. If margins were used, in all cases 80–125% or narrower margins were used. The narrower margins were likely to have been chosen to fit the efficacy equivalence margins in the phase III trials. For example, in the EPAR for Epoetin Alfa Hexal/Abseamed/Binocrit, it is stated that: ‘the acceptance range was then changed to 97–103% by protocol amendment based on haemoglobin (Hb) concentration changes defined as equivalence margins in phase III studies’.

In a way similar to the PK assessment, it is possible to achieve a positive opinion from the CHMP, even though not all PD parameters are found completely within the equivalence margins. For example, only one out of five relevant PD studies for Abasaglar met all criteria. The sponsor explained this by reference to the small sample sizes used and the presence of outliers that caused the criteria not to be met.

In the efficacy assessment in the phase III trials, in most cases there is one chosen primary endpoint, which is well defined in the EPAR. The only exceptions were Silapo/Retacrit, where two primary endpoints were used and Omnitrope, with four different primary endpoints. In both cases, no adjustment for multiplicity was mentioned. For Zarzio/Filgrastim Hexal, again, two efficacy endpoints were used but efficacy was not the primary endpoint in the phase III studies because it was a single‐arm trial with only comparison with historical data.

The chosen endpoints vary according to the indication. The observed types of endpoint were binary, continuous and count data, with continuous and count data being the most common (used nine and eight times, respectively) and binary data being less frequently used (four times). The different types of data also lead to different statistical models. For example, the predominantly used endpoint for biosimilars to filgrastim is the count of the number of days during the first cycle of chemotherapy, when neutropenia is severe. Methods of analysis for such an endpoint include standard approaches, such as Poisson regression 68. For the biosimilar to infliximab, it was assessed if the patients were responders or nonresponders, which is a binary variable. A responder is here defined according to the American College of Rheumatology 20% improvement criteria (ACR20) endpoint 69, which is one of the recommended scores by the EMA for the assessment of rheumatoid arthritis 70.

Obviously, the statistical methodology used has to be adapted to the chosen endpoint. However, even for biosimilars referring to the same reference product, the statistical model does not have to be the same. This is the case for Ovaleap and Bemfola, which are both biosimilars to follitropin alfa. For Ovaleap, a zero‐inflated Poisson (ZIP) regression model 71, with adjustment for age and country, was used as the primary analysis. The primary analysis for Bemfola was, depending on the data, either a Mann‐Whitney two one‐sided test (TOST) and a Bootstrap‐T for non‐normal data or a Schuirmann's TOST test 72. In the related product‐specific guidelines, it is stated that: ‘it should be taken into account that over‐stimulation as well as understimulation can result in cycle cancellation and a number of zero oocytes retrieved (primary endpoint)’ 73. Both approaches take this into account, so they seem to be in line with the guidelines, although the approach for Bemfola only considers the number of zero oocytes indirectly. Therefore, the ZIP model might be closer to the guidelines.

For biosimilars with the same reference product, in most cases identical endpoints were used. One exception is the substance epoetin alfa/zeta, for which, for the product Silapo/Retacrit, the mean dosage and the mean Hb level were used. In the efficacy assessment for the approval of Epoetin Alfa Hexal/Abseamed/Binocrit, the absolute change in Hb level was the primary endpoint. The reason for that might be that the studies were potentially planned before the first product‐specific guideline 63 was published. Therefore, the process was probably less standardized.

The equivalence margins that were used also depended on the indication and the chosen endpoint. Often, neither the publication nor the EPAR offer any explanation for the choice of margins. For biosimilars referring to the same reference product and with the same endpoint, the chosen margins were identical, except for Ovaleap and Bemfola, for which a minor deviation was observed: (−3, 3) and (−2.9, 2.9), respectively, were used. The difference is negligible but it shows that the decision regarding which equivalence margins to use is done individually for each sponsor.

## Discussion

The approval of biosimilars is more complex than for generics. Extensive clinical trials must be undertaken in order to prove the therapeutic equivalence of a biosimilar before the product can be authorized. This particularly includes analysis of PK and PD parameters and efficacy comparisons in phase III trials.

The present literature review of the EPARs published by the EMA and additional publications related to clinical trials shows that there is a large variability between the submitted applications. For example, the overall sample size in PK/PD trials varies considerably, from 24 to 269 subjects. In phase III trials, between 120 and 1295 patients were used. This variability can be partially explained by the different characteristics of the reference products.

In addition, the different types of endpoint in the efficacy assessment make it difficult to standardize the assessment method. For some products, binary data were used, whereas for other drugs the endpoint was continuous. This leads to different statistical models and methods, which need to be negotiated with the authorities. These different types of endpoint make it impossible to define general equivalence margins as these margins have to fit the therapeutic range of the drug. Interestingly, even for biosimilars with the same reference product, the endpoints, statistical model and equivalence margins, and also the sample size, were not in all cases comparable. This shows that there seems to be some flexibility for the sponsor on the decision as to how best to conduct the development plan.

The recommendations in the product‐specific guidelines were mostly followed. In some cases, there were also deviations. However, the product was approved in the end. It therefore seems to be possible to get approval for a product without following all regulatory guidelines in the assessment, as long as the overall application is convincing.

We believe that the EPARs should offer detailed information on the approval process, in order to make the decisions more transparent. Nonetheless, there are some aspects that could be further improved from our point of view. Firstly, the reports do not seem to be standardized. The length varies enormously; the shortest EPAR was published for Omnitrope (25 pages), whereas the longest was for Inflectra/Remsima (105 pages). The depth of information also differs; in some reports, the studies are described in detail, whereas in others, not even the trial doses are given. Furthermore, there is no unified structure, which makes it difficult to quickly identify the relevant information in the trial. For example, it would be easier to find information on the PK/PD trials if the details of the trial were summarized in an overview table, as is done in some of the newer EPARs for efficacy assessment (e.g. Bemfola). Secondly, in many cases no explanation is offered for the choice of equivalence margins in phase III trials. As the margins are crucial for the success of the trial, it would be desirable to understand the reasons for them. In addition, the EudraCT number that was introduced in 2004 can simplify the search for information about a specific trial. However, this number is not a part of the EPAR and, moreover, the database search for this number is complicated and often not successful. This makes it difficult to link the results in the EPARs to the more detailed information about a specific clinical trial in the database.

## Conclusions

There is high variability between the submitted applications. This is partially explainable by the different reference products, but sponsors can also negotiate the biosimilar development package with the regulatory agency. This was confirmed by the observation that, even for biosimilars with the same reference product, the relative weight put on PK/PD and phase III trials, the endpoints and the statistical models vary. Furthermore, it seems to be possible to get approval even if not all aspects in the overarching guidelines and the product‐specific guidelines are followed. It is also possible to achieve a opinion of the regulators if the equivalence criteria are not fulfilled. By contrast, it can be necessary to provide further justification, even though all equivalence criteria have been met. In conclusion, it seems that there is a fair degree of flexibility in the packages that sponsors can conduct to gain regulatory approval for a biosimilar. We note that some concerns on the part of the sponsor can be addressed by seeking early scientific advice from the EMA when planning the trials 74.

## Competing Interests

There are no competing interests to declare.

*Johanna Mielke was supported by the Swiss State Secretariat for Education, Research and Innovation (SERI) under contract number 999754557. The opinions expressed and arguments employed herein do not necessarily reflect the official views of the Swiss Government. The project is part of the IDEAS European training network (* http://www.ideas‐itn.eu */) from the European Union's Horizon 2020 research and innovation programme under the Marie Sklodowska‐Curie grant agreement No 633567. Bernd Jilma was supported by the Austrian Science Fund SFB54‐P04*.

## Supporting information


**Table S1** Summary of indications of reference product and biosimilars.

Supporting info item
