# Supplementary material for: Clinical trials for authorized biosimilars in the European Union: a systematic review
Source: Br J Clin Pharmacol. 2016 Sep 5;82(6):1444–57. doi: 10.1111/bcp.13076 (PMC5099555; doi:10.1111/bcp.13076)
Supplement: Supplementary file 1 — Supporting info item [file BCP-82-1444-s001.docx]

| **Reference Product (Company)** | **Indications** | **Biosimilar** | **Indications in studies** | **Indication applied for** |
| --- | --- | --- | --- | --- |
| Eprex  (Janssen) | EPREX is indicated for the treatment of patients with symptomatic or transfusion requiring anaemia associated with chronic renal failure to improve their quality of life by improving energy levels, exercise performance, fatigue and sleep patterns and by reducing the need for blood transfusions.  EPREX is also indicated for the treatment of anaemia and reduction of transfusion in patients with non-myeloid malignancies where anaemia develops as a result of concomitantly administered chemotherapy.  EPREX is also indicated in adult patients with mild-to-moderate anaemia (haemoglobin > 100 to ≤ 130 g/L) scheduled for elective surgery with an expected moderate blood loss (2 – 4 units or 900 to 1800 mL) to reduce exposure to allogeneic blood transfusion and to facilitate erythropoietic recovery.  EPREX is also indicated to augment autologous blood collection and to limit the decline in haemoglobin in anaemic adult patients who are scheduled for major elective surgery and who are not expected to pre-deposit their complete peri-operative blood needs. [1] | Silapo/Retacrit | correction of haemoglobin concentration in anaemic patients with end-stage renal failure on chronic  haemodialysis.  maintaining the haemoglobin concentration in these patients with renal anaemia | − Treatment of anaemia associated with chronic renal failure in adult and paediatric patients on  haemodialysis and adult patients on peritoneal dialysis.  − Treatment of severe anaemia of renal origin accompanied by clinical symptoms in adult patients  with renal insufficiency not yet undergoing dialysis.  − Treatment of anaemia and reduction of transfusion requirements in adult patients receiving  chemotherapy for solid tumours, malignant lymphoma or multiple myeloma, and at risk of  transfusion as assessed by the patient's general status (e.g. cardiovascular status, pre-existing  anaemia at the start of chemotherapy).  − SB309 can be used to increase the yield of autologous blood from patients in a predonation  programme. Its use in this indication must be balanced against the reported risk of  thromboembolic events. Treatment should only be given to patients with moderate anaemia  (haemoglobin (Hb) 10-13 g/dl [6.2-8.1 mmol/l], no iron deficiency), if blood saving procedures  are not available or insufficient when the scheduled major elective surgery requires a large  volume of blood (4 or more units of blood for females or 5 or more units for males).  The claimed indications initially included also reduction of allogeneic blood transfusions in adult  non-iron deficient patients prior to major elective orthopaedic surgery. However, during the CHMP  scientific assessment it became evident that efficacy and safety of Epoetin zeta have not been  demonstrated for the SC route of administration in immunocompetent patients. Therefore, the  applicant withdrew all indications using exclusively the SC route of administration in  immunocompetent patients. |
|  |  | Epoetin Alfa Hexal/ Abseamed/  Binocrit | CRF patients on haemodialysis  treatment of chemotherapy-associated anaemia | All the indications approved for the reference product, with the exception of the indication for increasing the yield of autologous blood from patients in a predonation programme. |
| Neupogen  (Amgen) | - Decrease the incidence of infection‚ as manifested by febrile neutropenia‚ in patients with nonmyeloid malignancies receiving myelosuppressive anticancer drugs associated with a significant incidence of severe neutropenia with fever  - Reduce the time to neutrophil recovery and the duration of fever, following induction or consolidation chemotherapy treatment of patients with acute myeloid leukemia (AML)  - Reduce the duration of neutropenia and neutropenia-related clinical sequelae‚ e.g.‚ febrile neutropenia, in patients with nonmyeloid malignancies undergoing myeloablative chemotherapy followed by bone marrow transplantation (BMT)  - Mobilize autologous hematopoietic progenitor cells into the peripheral blood for collection by leukapheresis  - Reduce the incidence and duration of sequelae of severe neutropenia (e.g.‚ fever‚ infections‚ oropharyngeal ulcers) in symptomatic patients with congenital neutropenia‚ cyclic neutropenia‚ or idiopathic neutropenia  - Increase survival in patients acutely exposed to myelosuppressive doses of radiation (Hematopoietic Syndrome of Acute Radiation Syndrome) [2] | Zarzio/  Filgrastim Hexal | chemotherapy-naïve breast cancer patients receiving doxorubicin and docetaxel chemotherapy and filgrastim as primary prophylaxis of  severe neutropenia | Same as reference product |
|  |  | Tevagrastim/  Ratiograstim/  Biograstim | Prophylaxis of severe neutropenia in  CTX-naïve patients with aggressive NHL (non-Hodgkin’s lymphoma) undergoing CTX  lung cancer (either small cell or non-small cell lung cancer) patients | - Tevagrastim is indicated for the treatment of persistent neutropenia (ANC less than or equal to 1.0 x 109/l) in patients with advanced HIV infection, in order to reduce the risk of bacterial infections when other options to manage neutropenia are inappropriate.  - In patients, children or adults, with severe congenital, cyclic, or idiopathic neutropenia with an absolute neutrophil count (ANC) of 0.5 x 109/l, and a history of severe or recurrent infections, long term administration of Tevagrastim is indicated to increase neutrophil counts and to reduce the incidence and duration of infection-related events.  - Tevagrastim is indicated for the mobilisation of peripheral blood progenitor cells (PBPC).  - Tevagrastim is indicated for the reduction in the duration of neutropenia and the incidence of febrile neutropenia in patients treated with established cytotoxic chemotherapy for malignancy (with the exception of chronic myeloid leukaemia and myelodysplastic syndromes) and for the reduction in the duration of neutropenia in patients undergoing myeloablative therapy followed by bone marrow transplantation considered to be at increased risk of prolonged severe neutropenia. The safety and efficacy of filgrastim are similar in adults and children receiving cytotoxic chemotherapy. |
|  |  | Nivestim | subjects receiving  doxorubicin and docetaxel as combination therapy for breast cancer | - Filgrastim is indicated for the treatment of persistent neutropenia (ANC less than or equal to  1.0 x 109 /l) in patients with advanced HIV infection, in order to reduce the risk of bacterial  infections when other options to manage neutropenia are inappropriate.  - In patients, children or adults, with severe congenital, cyclic, or idiopathic neutropenia with an  absolute neutrophil count (ANC) of ≤ 0.5 x 109 /l and a history of severe or recurrent infections,  long term administration of filgrastim is indicated to increase neutrophil counts and to reduce  the incidence and duration of infection-related events.  - Filgrastim is indicated for the mobilisation of peripheral blood progenitor cells (PBPC).  - Filgrastim is indicated for the reduction in the duration of neutropenia and the incidence of  febrile neutropenia in patients treated with established cytotoxic chemotherapy for malignancy  (with the exception of chronic myeloid leukaemia and myelodysplastic syndromes) and for the reduction in the duration of neutropenia in patients undergoing myeloablative therapy followed  by bone marrow transplantation considered to be at increased risk of prolonged severe  neutropenia. |
|  |  | Grastofil/Accofil | patients receiving chemotherapy known to induce neutropenia | - Grastofil is indicated for the reduction in the duration of neutropenia and the incidence of febrile neutropenia in adult patients treated with established cytotoxic chemotherapy for malignancy (with the exception of chronic myeloid leukaemia and myelodysplastic syndromes) and for the reduction in the duration of neutropenia in adult patients undergoing myeloablative therapy followed by bone marrow transplantation considered to be at increased risk of prolonged severe neutropenia.  - Grastofil is indicated for the mobilisation of peripheral blood progenitor cells (PBPCs) in adults  - In adult patients with severe congenital, cyclic, or idiopathic neutropenia with an absolute neutrophil count (ANC) of ≤ 0.5 x 109/L, and a history of severe or recurrent infections, long term administration of Grastofil is indicated to increase neutrophil counts and to reduce the incidence and duration of infection-related events.  - Grastofil is indicated for the treatment of persistent neutropenia (ANC less than or equal to 1.0 x 109/L) in adults with advanced HIV infection, in order to reduce the risk of bacterial infections when other options to manage neutropenia are inappropriate. |
| Gonal-f  (Merck) | - adult women (aged 18 years or over) who do not produce eggs and do not respond to treatment with clomiphene citrate (another medicine that stimulates the ovaries to produce eggs); - adult women who are undergoing assisted reproductive techniques (fertility treatment) such as in-vitro fertilisation. GONAL-f is given to stimulate the ovaries to produce more than one egg at a time; - adult women with severe deficiency (very low levels) of luteinising hormone (LH) and follicle stimulating hormone (FSH). GONAL-f is given together with a medicine containing LH to stimulate the eggs to mature in the ovaries; - adult men who have hypogonadotrophic hypogonadism (a rare hormone deficiency disease). GONAL-f is used together with human chorionic gonadotrophin (hCG) to stimulate sperm production. [3] | Oveleap | Stimulation of multifollicular development in patients undergoing superovulation for ART. | Same as reference product |
|  |  | Bemfola | Stimulation of multifollicular development in patients undergoing superovulation for ART. | Same as reference product |
| Lantus  (Sanofi-Aventis) | LANTUS is a long-acting human insulin analog indicated to improve glycemic control in adults and pediatric patients with type 1 diabetes mellitus and in adults with type 2 diabetes mellitus. [4] | Absaglar | Treatment of type 1 and type 2 diabetes mellitus | Same as reference product. |
| Genotropin  (Pfizer) | Pediatric: Treatment of children with growth failure due to growth hormone deficiency (GHD), Prader-Willi syndrome, Small for Gestational Age, Turner syndrome, and Idiopathic Short Stature  Adult: Treatment of adults with either adult onset or childhood onset GHD [5] | Omnitrope | Prepubertal, GH-deficient patients with a stature of <-2 SDS for chronological age, and a spontaneous growth rate of <-1 SDS over an interval of at least six months prior to enrolment | - Growth disturbance due to insufficient secretion of growth hormone and growth disturbance associated with Turner syndrome or chronic renal insufficiency.  - Growth disturbance (current height SDS < -2.5 and parental adjusted SDS < -1) in short children born small for gestational age (SGA), with a birth weight and/or length below -2 SD, who failed to show  catch-up growth (HV SDS < 0 during the last year) by 4 years of age or later  - Prader-Willi syndrome (PWS), for improvement of growth and body composition. The diagnosis of PWS should be confirmed  by appropriate genetic testing.  - Replacement therapy in adults with pronounced growth hormone  deficiency. |
| Enbrel (Pfizer) | Enbrel is used in the following diseases:   - moderate to severe rheumatoid arthritis - certain forms of juvenile idiopathic arthritis - psoriatic arthritis in adults who have not responded adequately to other treatments - severe ankylosing spondylitis plaque psoriasis (a disease causing red, scaly patches on the skin) in adults - Severe non-radiographic axial spondyloarthritis [6] | Benepali |  | Treatment of rheumatoid arthritis, psoriatic arthritis, ankylosing  spondylitis, non-radiographic axial spondyloarthritis and plaque psoriasis in adult patients.  (no approval for the paediatric indications that Enbrel is used for) |
| Remicade  (Janssen) | - rheumatoid arthritis (an immune system disease causing inflammation of the joints). Remicade is used with methotrexate (a medicine that acts on the immune system); - -Crohn’s disease (a disease causing inflammation of the digestive tract), when the disease is moderate to severe or fistulising (with the formation of fistulae, abnormal passageways between the gut and other organs); - ulcerative colitis (a disease causing inflammation and ulcers in the lining of the gut); - ankylosing spondylitis (a disease causing inflammation and pain in the joints of the spine); - psoriatic arthritis (a disease causing red, scaly patches on the skin and inflammation of the joints); - psoriasis (a disease causing red, scaly patches on the skin). [7] | Remsima/  Inflectra | patients with active   \| Rheumatoid arthritis \| \| --- \|   when co-administrated with methotrexate;  patients with ankylosing spondylitis | Same as reference product |
|  |  | Flixabi | Subjects with moderate to severe rheumatoid arthritis despite methotrexate therapy. | Same as reference product |

**Table S: Summary of indications of reference product and biosimilars.**

References:

[1] Janssen. Eprex Intravenous & Subcutaneous Injection: product information. 2015. Available at <http://www.janssen.com/australia/sites/www_janssen_com_australia/files/product/pdf/eprex_pi.pdf> (last accessed: 10 Jan 2016).

[2] Amgen. Neupogen: highlights of prescribing information. 2015. Available at <http://pi.amgen.com/united_states/neupogen/neupogen_pi_hcp_english.pdf> (last accessed: 10 Jan 2016).

[3] CHMP. Gonal-f: EPAR summary for the public. 2009. Available at <http://www.ema.europa.eu/docs/en_GB/document_library/EPAR_-_Summary_for_the_public/human/000071/WC500023742.pdf> (last accessed: 10 Jan 2016).

[4] Sanofi. Lantus: highlights of prescribing information. 2015. Available at <http://products.sanofi.us/lantus/lantus.pdf> (last accessed: 10 Jan 2016).

[5] Pfizer. Genotropin: highlights of prescribing information. 2015. Available at <http://labeling.pfizer.com/ShowLabeling.aspx?id=577> (last accessed: 10 Jan 2016).

[6] CHMP. Enbrel: EPAR summary for the public. 2014. Available at URL <http://www.ema.europa.eu/docs/en_GB/document_library/EPAR_-_Summary_for_the_public/human/000262/WC500027364.pdf> (last accessed: 26 Feb 2016).

[7] CHMP. Remicade: EPAR summary for the public. 2010. Available at <http://www.ema.europa.eu/docs/en_GB/document_library/EPAR_-_Summary_for_the_public/human/000240/WC500050883.pdf> (last accessed: 10 Jan 2016).
